# Transcriptome and Resistance-Related Genes Analysis of *Botrytis cinerea* B05.10 Strain to Different Selective Pressures of Cyprodinil and Fenhexamid

**DOI:** 10.3389/fmicb.2018.02591

**Published:** 2018-10-30

**Authors:** Xuegui Wang, Changwei Gong, Yun Zhao, Litao Shen

**Affiliations:** Biorational Pesticide Research Lab, Sichuan Agricultural University, Chengdu, China

**Keywords:** *Botrytis cinerea*, cyprodinil, fenhexamid, transcriptome analysis, mixed-functional oxidase

## Abstract

The pathogen *Botrytis cinerea* is a very dangerous pathogen that infects many economically important crops such as grape, strawberry, tomato, and eggplant. Cyprodinil, a pyrimidine amine fungicide, and fenhexamid, an amide fungicide, are new reagents for controlling gray mold with special efficacy. It is necessary to understand the change trends in the toxicological and physiological characteristics of *B. cinerea* with successive selective pressures of cyprodinil and fenhexamid to elongate the serving life of these fungicides for effective disease control. The toxicities of cyprodinil and fenhexamid at successive concentrations of EC_25_, EC_50_ and EC_75_ on *B. cinerea* strain BO5.10 were assayed along with mycelial growth-inhibition capacity. The results showed that the EC_50_ value of the cyprodinil-treated F_27_ strain increased approximately 18-fold, whereas of which in the fenhexamid-treated F_27_ strain increased only 3-fold compared with that of the F_0_ strain. The conductivities and glycerinum contents of the strains resistant to cyprodinil and fenhexamid were obviously enhanced; in contrast, the oxalic acid contents were decreased compared with those in the F_0_ strain. The transcriptomes of the F_27_ control (T01), cyprodinil-treated (T02) and fenhexamid- treated (T03) strains were analyzed, and the expression levels of functional genes in the T02 and T03 strains were significantly increased compared with those in the T01 strain; these results were further validated using qRT-PCR. The results indicated that the relative expression of two genes encoding mixed-functional oxidases (MFOs) *BC1G_16062* and *BC1G_16084*, two genes encoding transmembrane proteins *BC1G_12366* and *BC1G_13768*, two genes encoding Zinc finger proteins *BC1G_13764* and *BC1G_10483*,one gene encoding citrate synthase enzyme *BC1G_09151*, one gene encoding gluconolactonase *BC1G_15612* in the T02 and T03 strains and one gene encoding lysophospholipids enzyme *BC1G_04893* in the T3 strain increased substantially compared with that in the T1 strain (*P* < 0.01). Functional prediction analysis of upregulated gene expression and structural verification was also performed, and the results showed that *BC1G_10483* was a ZnF_C2HC transcriptional regulator interacting with the Sp1 element of these genes to respond to the pressures from cyprodinil and fenhexamid. Our results could contribute to a better understanding of the resistance mechanism of *B. cinerea* against cyprodinil and fenhexamid.

## Introduction

*Botrytis cinerea* is an aggressive plant disease and more than 200 plant species, such as tomato, grape, strawberry and so on, are infected resulting in large output loss (Shao et al., 2015). At present, the control of *B. cinerea* still mainly relies on chemical agents, with some other auxiliary methods, such as breeding disease-resistant varieties, rational fertilization and improving cultivation facilities. *B. cinerea* causes a disease with a high resistance risk (Jiang et al., 2009), and extensive use of the same types of fungicides for a long time has contributed to serious resistance, such as benzimidazole (carbendazol, benomyl) (Leroux et al., 2002), thiocarbamates (thiophanate) (Zhang et al., 2009), and dicarboxyl imide (Sansone et al., 2005; Sun et al., 2010) (iprodione, procymidone).

Due to the interactive resistance of antifungal mutant strains to fungicides, developing new fungicides has become increasingly difficult. The effective control methods for graymold developed in recent years include pyrimidine amine (cyprodinil), pyrrole (fludioxonil), and amides (fenhexamid). It is necessary to assay the biological activity and to evaluate the resistance risk before widely using new fungicides in order to delay the development of *B. cinerea* resistance against those newer fungicides to elongate their service time. A few cases have demonstrated that there was some cross-resistance risk even though their structural classes are significant difference. For example, the resistant-dicarboximides strains of *B. cinerea* also produced resistance to some fungicides, including dichloran, quintozene, biphenyl and so on, which maybe act on a same possible point, histidine kinase (Leroux et al., 2002).

Cyprodinil, a pyrimidine amine fungicide acting as an inhibitor of the biosynthesis of methionine, was developed by Novartis (Basel, Switzerland). The water-dispersible granule has been known since 2000 to provide protection, treatment, internal suction and transmission in the root and leaf (Bernardo and René, 2012). Cyprodinil has low toxicity, inhibits the secretion of the extracellular protease hydrolysis enzyme of *B. cinerea* and pathogen penetration, interferes with the fungal life cycle and destroys mycelium growth and development in plants (Mofeed and Mosleh, 2013). There is no cross-resistance among the fungicides benzimidazoles, thiocarbamate, carbamate, imidazoles and so on because of their different structures and target points.

Fenhexamid, an amide fungicide acting on sterol synthesis, was developed by Bayer Crop Science Co., Ltd., (Leverkusen, Germany) and displayed good efficacy against grape gray mold (Zocco et al., 2008). Fenhexamid can inhibit bacterial growth, such as by decreasing the growth of mycelium and inhibiting the extension of the bud tube and the conidia germination (Debieu et al., 2001). Fenhexamid has characteristics of biodegradability, low toxicity, environmental friendliness and no cross-resistance with the production of pyrimidine, carbamate, pyrrole, pyridine or other fungicides widely applied in agriculture.

Currently, there are only a few reports on the field efficacy of cyprodinil and fenhexamid, and the physiological and biochemical effects on resistant strains have rarely been studied. In this study, we assay the toxicities, physiological and biochemical characteristics and transcription of the F_27_ strains of *B. cinerea* B05.10 continuously treated with cyprodinil and fenhexamid at concentrations of EC_25_, EC_50_ and EC_75_. The results are expected to promote the comprehension of the mechanisms of resistance development of *B. cinerea* against cyprodinil and fenhexamid and provide a foundation for the design of more effective resistance management strategies.

## Materials and Methods

### Fungicides and Strain

The technical fungicides cyprodinil and fenhexamid (a.i.98%) were purchased from Shan Dong Yi Jia Agricultural Chemical Co., Ltd., and the BO5.10 strain was kindly provided by Prof. Li Guoqing of the Plant Pathology Laboratory, Huazhong Agricultural University in January 2013.

### Toxicity Changes in the BO5.10 Strain to Different Pressures of Cyprodinil and Fenhexamid

The toxicities of cyprodinil and fenhexamid on the BO5.10 strain (F_0_) were assayed with mycelial growth inhibition as described by Kuang et al. (2011). Five concentrations were set for each fungicide. One milliliter of prepared solution was added to a sterile petri dish, followed by the addition of 9 ml of PDA and rapid shaking. After complete cooling, one 5-mm hyphal disk cut from a 3-day-old PDA culture was placed in the center of each plate. Each subsequent concentration increased two-fold, and 1 ml of distilled water was used as a control. Then, incubated those colonies about 72 h at 23°C in a biochemical incubator in the dark and measured their diameters to assess the inhibition of mycelial growth. The toxicity regression equation of the inhibition rate against the log_10_ concentrations of cyprodinil and fenhexamid was calculated though SAS PROC REG (version 9.1, SAS institute, Cary, NC). Inhibition rate (%) = (colony diameter of control treatment – colony diameter of fungicide treatment)/(colony diameter of control treatment – 5-mm hyphal disk diameter) (Zhou et al., 2016).

According to the toxicity regression of cyprodinil and fenhexamid on the BO5.10 strain (F_0_), the concentrations of EC_25_, EC_50_, and EC_75_ were calculated, and the BO5.10 strain (F_0_) was successively cultured in three replicates under the three concentrations thus generating the F_3_ strains. The effects of the EC_25_, EC_50_, and EC_75_ concentrations of cyprodinil and fenhexamid on the F_3_ strains were assayed with the same methodology, and additional screenings were continually preformed procedure until we obtained the F_27_ strains. Those strains with clearly enhanced toxicities were stored in a mixture of glycerinum-saline (1:3, v/v) at −20°C to investigate the resistance- related physiological and biochemical characteristics.

### Membrane Permeability and Glycerinum and Oxalic Acid Contents of the Strain With Significantly Enhanced Toxicity

Those strains with clearly enhanced toxicities were incubated in 100 ml of PD nutrient solution in 250 ml laboratory flasks (approximately five to seven 5-mm hyphal disks per flask), and then cultivation was performed in a constant-temperature shaker (23°C, 120 rpm). The BO5.10 strain was used as the blank control. After the tested strains had been cultivated for approximately 7 days, the hyphae were repeatedly flushed with deionized water and vacuum filtered for approximately 15 min. A 0.5-g sample for each treatment was added to 20 ml of double distilled water; the conductivities were detected at 0, 5, 10, 20, 40, 60, 80, 100, 120, 140, 160, and 180 min after treatment; and the final conductivities were measured via boiling the mycelium in water for 5 min. Three repetitions were performed for each treatment, and the relative conductivity was calculated as following: the conductivity of different times/the final conductivity (Li et al., 2015).

Glycerine copper colorimetry (Yan and Qiu, 2004; Duan et al., 2013) was used to assay the glycerinum contents of the hyphae under different treatments. Three kinds of solutions, including 1 ml of CuSO_4_ (0.05 g/l), 3.5 ml of NaOH (0.05 g/ml) and 10-ml of glycerinum in double distilled water (0, 0.0025, 0.003, 0.004, 0.005, 0.006, 0.008, 0.01, 0.015, and 0.02 g/ml) were transferred into different 50-ml flasks, respectively. Then quickly shook the mixtures, transferred into a 50-ml centrifuge tube, and centrifuged at 100 rpm for 12 min. The absorbance of the supernatant was recorded at 630 nm to establishing the standard curve. A 0.5-g sample from each treatment was ground with 20 ml of sterile water and some quartz sand. The supernatant was transferred into a 50-ml centrifuge tube, heated in an 80°C water bath for 15 min, and centrifuged at 12,000 × *g* for 10 min. The glycerinum contents in the supernatant were detected using distilled water as a control. All treatments were performed in triplicate.

The standard curve of oxalic acid was established as follows: 2 ml of FeCl_3_ solution (0.5 g/ml), 20 ml of HCl–KCl buffer solution (KCl 50 mM, pH = 2) and 1.2 ml of sulphosalicylic acid solution (0.5 mg/ml) were added to a 25-ml volumetric flask in order; then, aliquots of 0, 0.1, 0.2, 0.4, and 0.8 ml of sodium oxalate solution (2 mg/ml) were transferred into different 25-ml volumetric flasks, brought to volume with double-distilled water and shaken. The oxalic acid contents were determined based on changes in absorbance at 510 nm in a UV 2000- Spectrophotometer [Unic (Shang Hai) Instruments Incorporated] with double-distilled water as a control (Duan et al., 2014). The pretreatment of each treatment was the same as the approach when assaying for the glycerinum contents. The supernatant was transferred into a 50 ml centrifuge tube and centrifuged at 380 × *g* for 10 min. The absorbance values of the supernatant were recorded at 510 nm, and the oxalic acid contents were calculated with the standard curve.

### Transcriptome Analysis of the BO5.10 and Fungicide-Treated Strains

#### Library Construction and Sequencing

Total RNAs were extracted from F_27_ strains screened from BO5.10 (T01) with EC_50_ concentrations of fenhexamid (T02) and cyprodinil (T03) and BO5.10 (F_0_) using Trizol^®^ Reagent (Invitrogen^TM^) according to the manufacturer’s instructions and all following of library construction and sequencing were performed as the description of Wang et al. (2018). After the quality of total RNA was checked out, eukaryotic mRNA was enriched by Oligo(dT) beads, then the enriched mRNA was fragmented into short fragments using fragmentation buffer and reverse transcripted into cDNA with random primers. The second-strand cDNA were synthesized by DNA polymerase I, RNase H, dNTP and buffer. Then the cDNA fragments were purified with QiaQuik PCR extraction kit, end repaired, polys(A) added, and ligated to Illumina sequencing adapters. The ligation products were size selected by agarose gel electrophoresis, PCR amplified, and sequenced using IlluminaHiSeq^TM^ 4000.

### Sequence Alignment Between Transcriptome Data and Reference Genome Sequence

The high quality reads obtained in this study have been deposited in the NCBI SRA database (accession number: SUB4543717). Owing to reads obtained from the sequencing machines including dirty reads, reads containing more than 10% of unknown nucleotides, more than 50% of low quality (*Q*-value ≤ 10) bases and adapters were further filtered in order to get high quality clean reads. Clean reads of three samples were aligned with the reference genome^1^ through TopHat2, and the reference genome positions and the unique sequences of the tested samples were identified (Kim et al., 2013). Clean reads that were able to align to the reference genome were referred to as mapped reads.

### Statistics and Annotation of Single Nucleotide Polymorphisms

Based on the alignment results between the reads of the three treatments and the reference genome used by TopHat2, single erroneously paired nucleotides (SNPs) as determined by comparison with the reference genome were distinguished by The Genome Analysis Toolkit (GATK) (McKenna et al., 2010), and then, these SNP sites were analyzed by the program SnpEff (Cingolani et al., 2012). Furthermore, it was possible to analyze whether these SNP sites affect the gene expression level or encoded protein type.

### Discover New Gene and Expressed Gene

Based on the selected reference genome sequence, mapped reads were spliced though Cufflinks software (Trapnell et al., 2010) and compared with the original genome annotation to search for original transcription areas that were not annotated, and the species of transcription and new genes were explored to complement and complete the existing genome annotation information. The newly discovered genes were aligned using BLAST software (Altschul et al., 1997) in NR, Swiss-Prot, GO, COG, KOG, Pfam, ad KEGG databases, and the KEGG orthology (Ashburner et al., 2000; Tatusov et al., 2000; Apweiler et al., 2004; Koonin et al., 2004; Deng et al., 2006; Finn et al., 2013; Kanehisa et al., 2004) results of new genes were generated using KOBAS2.0 (Xie et al., 2011). Information on new gene annotation was acquired by comparison between HMMER software and the Pfam database after the amino acid sequences of new genes had been predicted.

Transcriptome sequencing can be modeled as a random sampling process, that is, from an arbitrary sample of the transcriptome is generated a nucleic acid sequence of an independent random sequence fragment. The related to the mapped data, the length of the transcribed sequence and the transcription expression levels were used to determine a fragment number that truly reflected the transcript expression level, the number of mapped reads and the normalized length of transcription. Cuffquant and Cuffnorm used Fragments Per Kilobase of transcript per Million fragments mapped (FPKM) as a measure of transcription or gene expression levels (Florea et al., 2013; Lei et al., 2014) as follows:

$$\mathrm{FPKM}=\frac{\mathrm{cDNA fragements}}{\mathrm{mapped fragements (Millions)}\times \mathrm{transcript length(kb)}}$$

In the formula, cDNA fragments stand for the number of segments that were able to align on a transcript, that is, the number of two read pairs; mapped fragments (millions) represent the total number of segments that were able to align during transcription, in a denomination of 10^∧^6 bp; and transcript length (kb) represents the length of transcription, in a denomination of 10^∧^3 bp.

### Differential Expression Analysis

The expressed genes of three samples (T01, T02, and T03) were analyzed using EBSeq. Because differential expression analysis of the transcriptome sequencing was performed independently on a large, statistically hypothetical set of gene expression values, some results could be false positives. Therefore, the significant *P*-values extracted from the original hypothesis testing were corrected using the recognized Benjamini-Hochberg correction method, and eventually, the fold change (the ratio of expression amount between two samples) and FDR (based on correcting the significant *P*-values) were used as the key indicators of screening differentially expressed genes (DEGs), with Fold Change ≥ 2 and FDR < 0.01 as screening criteria. According to the requirements of each group, a Venn diagram of DEGs was drawn and analyzed by hierarchical clustering and grouped genes with the same or similar expression patterns. The DEGs were aligned using BLAST software in the COG and KEGG databases, and annotated with KOBAS2.0 and aligned using BLAST software in the STRING database (a database of multiple species hypothetic and experimental protein-protein interactions), and then, homologous proteins were searched for. The protein-protein interaction networks were built based on the interactions of homologous proteins (Franceschini et al., 2013), and a protein-protein interaction network scheme was drawn using Cytoscape software for visualization (Shannon et al., 2003).

### Quantitative PCR (qRT-PCR)

Total RNA from the F_27_ strains screened from BO5.10 treated with the EC_50_ concentrations of fenhexamid (T02) and cyprodinil (T03) and from BO5.10 (T01) was extracted as described in section of Library Construction and Sequencing. For the reverse transcription reaction, the synthesis of First-Strand cDNA was used according to the kit. Then, the cDNA was stored at −20°C for qRT-PCR.

Sixteen pairs of respective primers, including the cDNAs of fifteen genes (*BC1G_12765, BC1G_12768, BC1G_16062, BC1G_16084, BC1G_04656, BC1G_04779, BC1G_12366, BC1G_13768, BC1G_04893, BC1G_09151, BC1G_15612, BC1G_02313, BC1G_04879, BC1G_13764, BC1G_10483*) and β_*tubulin* using for a house-keeping gene (Yang et al., 2012) in the T1, T2 and T3 strains were displayed in Supplementary Table 1 for amplifying by qRT-PCR, which was a total 20 μl reaction system, including 1 μl of cDNA, 0.4 μl of each primer, 10 μl of 2 × TransStart^®^ TopTaq Green qPCR SuperMix (Transgene, Beijing, China) and 8.2 μl of double-distilled water. The standard cycle was performed in 94°C for 30 s, 40 cycles of 94°C for 30 s and 60°C for 30 s. The initial temperature of the dissolution curve was 68°C. The β_*tubulin* gene of *B. cinerea* was used as the internal control. The relative normalized expression of target genes was calculated using the 2^−ΔΔCt^ method (Livak and Schmittgen, 2001). Three independent replicates were held in all strains and target genes, and all data were analyzed via one-way ANOVA by PROC GLM of the SAS program. All means were compared by least squared difference (LSD) tests at Type I error = 0.05.

### Function Prediction of Upregulated Genes and Structural Verification

The structural domains of the upregulated genes that were annotated using GO annotation were predicted to be P450^2^, their structural domains were verified using motif_scan and ProDom^3^, and their topological domains were analyzed^4^.

The *cis*-acting elements of the DEGs were predicted using AliBaba2.1^5^, and their *trans*-acting factors, which could combine with the *cis*-acting elements through JASPAR CORE Fungi^6^, were analyzed. Prediction analysis of transcription factors of DEGs was performed through a BLAST Search of FTFD^7^, and their structural domains were verified using SMART^8^ and the PFAM SEARCH^9^.

## Results

### Toxicity Changes in the BO5.10 Strain in Response to Different Concentrations of Cyprodinil and Fenhexamid

**FIGURE 1:**
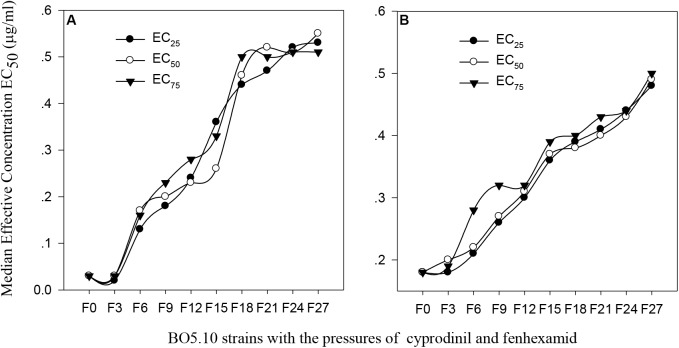
Change of virulence of *B. cinerea* to the different concentration of cyprodinil and fenhexamid. **(A)** Cyprodinil; **(B)** fenhexamid.

The original EC_50_ value of cyprodinil on the BO5.10 strain (F_0_) was 0.03 μg/ml, and those in the F_6_ strains generated through successively screening with the EC_25_, EC_50_ and EC_75_ concentrations increased to 0.13 μg/ml, 0.17 μg/ml and 0.16 μg/ml, respectively, and the toxicities of these strains were increased almost 5-fold. The toxicities of strains F_9_ to F_15_ screened with the EC_25_, EC_50_ and EC_75_ concentrations slightly increased, whereas the toxicities of F_18_ strains rapidly increased under the pressures of EC_50_ (0.50 μg/ml); the toxicities plateaued after 20 screenings. Finally, the EC_50_ values of cyprodinil on the F_27_ strain screened with the EC_25_, EC_50_ and EC_75_ concentrations reached 0.53 μg/ml, 0.55 μg/ml, and 0.51 μg/ml, respectively, and increased almost 18-fold compared with that of the BO5.10 strain (F_0_) (Figure 1A).

The toxicity of fenhexamid on the BO5.10 strain (F_0_) continuously increased as the EC_25_ concentration pressure increased from strains F_0_ to F_27_, which was enlarged approximately 3-fold compared with that of the F_0_ strain (0.18 μg/ml). The toxicity of the strains screened with the EC_50_ concentration from strains F_0_ to F_15_ rapidly increased, followed by a slim change from strains F_15_ to F_18_, with EC_50_ values from 0.37 to 0.49 μg/ml. The toxicity of the strains screened with EC_75_ from strains F_0_ to F_9_ significantly increased. The trend was slow from strains F_9_ to F_15_, whereas the toxicities of F_15_ to F_27_ strains continuously increased with EC_50_ values from 0.39 μg/ml to 0.50 μg/ml (Figure 1B).

### Membrane Permeability and Glycerinum and Oxalic Acid Contents of BO5.10 Under the Successive Pressure of Cyprodinil and Fenhexamid

**FIGURE 2:**
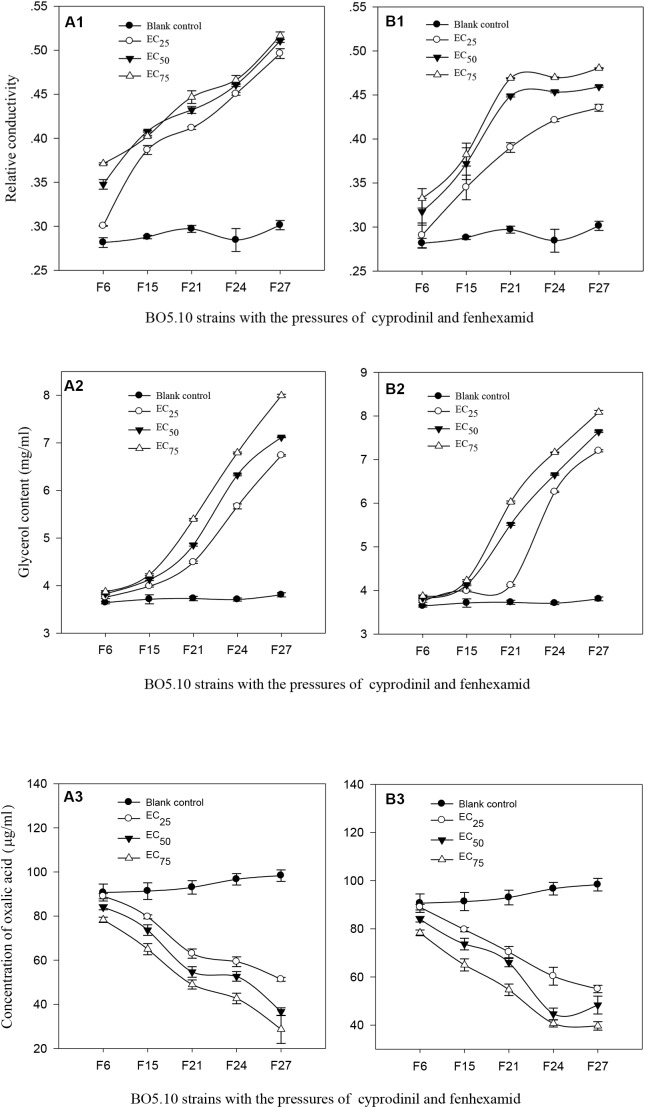
**(A1,B1)** Relative conductivity, **(A2,B2)** content of glycerol, and **(A3,B3)** oxalic acid of *B. cinerea* to different concentration of cyprodinil **(A)** and fenhexamid **(B)**. The treatments of Blank control, EC_25_, EC_50_ and EC_75_ represents the strains which have been preformed successively 27 screenings with the concentrations of EC_25_, EC_50_ and EC_75_ of cyprodinil **(A)** and fenhexamid **(B)** on the BO5.10, and the toxicities of the strains F_6_, F_21_, F_24_, F_27_ were clearly decreased compared with the BO5.10 strain on cyprodinil **(A)** and fenhexamid **(B)**, then we detected their relative conductivity, content of glycerol and oxalic acid.

The results indicated that the relative conductivities of BO5.10 from strains F_6_ to F_27_without fungicidal pressure were 0.2816∼0.3014 (*P* > 0.05), whereas which in the strains screened with the EC_25_, EC_50_, and EC_75_concentrations of cyprodinil from strains F_6_ to F_27_ increased, reaching 0.3005∼0.4962, 0.3477∼0.5106 and 0.3712∼0.5165, respectively, and were significantly higher than that in the blank control (*P* < 0.01) (Figure 2A1). The relative conductivities of strains F_6_–F_27_ screened with different concentrations of fenhexamid were clearly enhanced, especially for strains F_6_–F_19_ and F_24_–F_27_ screened with the EC_50_ and EC_75_ concentrations, achieving 0.3174∼0.4489, 0.3326∼0.4691 and 0.4536∼0.4594, 0.4698∼0.4805, respectively, and were significantly different from those in the EC_25_ treatment (0.2906∼0.3903 and 0.4213∼0.4354) (*P* < 0.05). The relative conductivities of strains F_6_–F_27_ screened with the EC_25_, EC_50_, and EC_75_ concentrations of fenhexamid were significantly stronger than those in the control (*P* < 0.01) (Figure 2B1).

The glycerinum contents in those strains screened with fungicides were positively correlated with the screen dosage, especially for strains F_19_–F_27_ screened with the EC_75_ of cyprodinil, which were remarkably enhanced (3.87∼7.99 mg/ml) and significantly different from the other treatments (*P* < 0.05) (Figure 2A2). The glycerinum contents of strains F_15_–F_27_ screened with the EC_75_ and EC_50_ of fenhexamid were conspicuously elevated (3.83∼7.64 and 3.87∼8.09 mg/ml) and were extremely higher than in the other treatments (*P* < 0.01), followed by strains F_19_–F_27_ screened with the EC_25_ of fenhexamid, reaching 3.75∼7.20 mg/ml, and were significantly different from those in the control (3.64∼3.81 mg/ml) (Figure 2B2).

The oxalic acid contents in the strains screened with fungicides were negatively correlated with the screen dosages, and those in the blank control were the highest, especially for strains F_6_–F_27_, with 90.67∼98.33 μg/ml. In contrast, the oxalic acid content of the F_27_ strain screened with the EC_75_ treatment of cyprodinil was the lowest (28.67 μg/ml) (*P* < 0.01), followed by those in the EC_50_ and EC_25_ treatments, reaching 84.0∼36.67 and 89.00∼59.33 μg/ml, respectively (Figure 2A3). The oxalic acid contents in strains F_6_–F_27_ screened with the EC_75_, EC_50_, and EC_25_ concentrations of fenhexamid reached 89.00∼55.00 μg/ml, 84.00∼48.33 μg/ml and 78.33∼39.67 μg/ml, respectively. Furthermore, those in the treatments with EC_75_ and EC_50_ were clearly lower than those in the treatment with the EC_25_ (*P* < 0.05) (Figure 2B3).

### Transcriptional Data Statistics and Sequence Alignment Between Transcriptome Data and the Reference Genome Sequence

**FIGURE 3:**
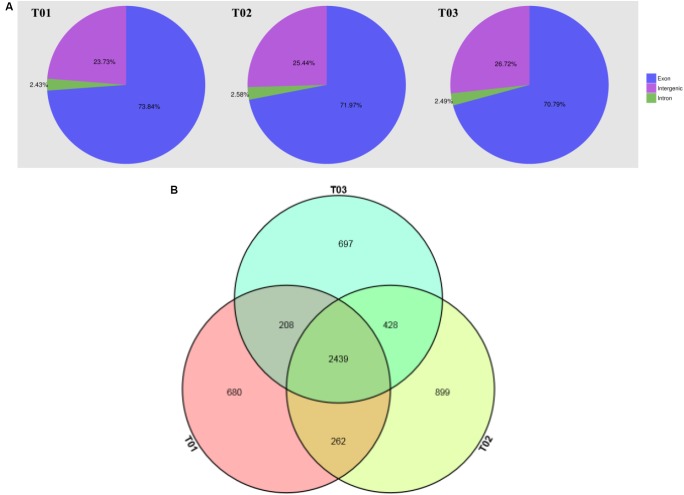
**(A)** Distribution of Reads in different regions of the genome. The genome is divided into exon, intergenic, and intron regions, and the region size is determined by the percentage of Mapped Reads to the corresponding region in all Mapped Reads. The blue region is reads in the exon region; The purple region represents reads in the intergenic region; The green region represents reads in the intron region. **(B)** The figure of Venn of samples. The magenta circle region indicates Single Nucleotide Polymorphisms that exists in T01; The pale yellow circle region indicates Single Nucleotide Polymorphisms that exists in T02; The pale blue circle region indicates Single Nucleotide Polymorphisms that exists in T03; The overlapping area indicates Single Nucleotide Polymorphisms presented in two or three strains.

The results indicated that these samples had nearly 13.79 Gb of clean data, and the Q30 exceeded 95.72% (Table 1). According to the results, the contrast efficiencies of reads reached 80.62∼82.96% compared to the reference genome (Table 2). Transcriptome sequencing of three samples was analyzed by Illumina HiSeqTM 4000, and more than 23,000,000 mapped reads were obtained after screening and filtration in each sample contracted by the reference genome. The ratios of mapped reads, uniquely mapped reads, and multiple mapped reads compared with the total reads reached over 80, 70, and 10%, respectively. The ratios of reads mapped to “+” and reads mapped to “−” were all over 35% (Figure 3A).

**Table 1:** Statistics of transcriptional data of samples.

Strain	BMK-ID	Clean reads	Clean bases	GC Content	% ≥ Q30
B05.10	T01	16,393,573	4,848,823,732	46.79%	95.77%
H1	T02	15,859,677	4,677,392,268	46.69%	95.72%
M1	T03	14,422,045	4,260,308,718	46.65%	96.12%

*T01, T02, and T03 stand for the BO5.10 strain with no fungicide treatment, F_27_ strains successively screened with EC_50_ concentration of cyprodinil or fenhexamid, respectively; BMK-ID, the sample number for analysis; Clean reads, the total of pair-end Reads in Clean Data; Clean bases, the total base Clean Data; GC content, the percent of G and C in total base for the Clean Data; ≥Q30%, the percent of quantity ≥30 in Clean Data.*

**Table 2:** Comparison of the data of tested sample and the reference genome.

BMK-ID	Total Reads	Mapped Reads	Uniq Mapped Reads	Multiple Map Reads	Reads Map to ′ + ′	Reads Map to ′−′
**T01**	32,787,146	26,434,570 (80.62%)	23,031,651 (70.25%)	3,402,919 (10.38%)	11,751,078 (35.84%)	11,715,474 (35.73%)
**T02**	31,719,354	26,130,916 (82.38%)	22,913,976 (72.24%)	3,216,940 (10.14%)	11,619,415 (36.63%)	11,621,229 (36.64%)
**T03**	28,844,090	23,929,866 (82.96%)	20,992,277 (72.78%)	2,937,589 (10.18%)	10,695,604 (37.08%)	10,697,777 (37.09%)

*BMK-ID, the sample number for analysis; Total Reads, the number of Clean Reads; Mapped Reads, the number of Clean Reads that were able to align on the reference genome data, and the ratio of the mapped reads compared with the total Reads; Uniq Mapped Reads, the number of Clean Reads that were able to align on the only location of the reference genome, and the ratio of the uniq mapped reads compared with the total Reads; Multiple Map Reads, the number of Clean Reads that were able to align on the multiple location of the reference genome, and the ratio of the multiple mapped reads compared with the total Reads; Reads Map to “+”, the number of Clean Reads that were able to align on the positive-sense strands of the reference genome data, and the ratio of these reads compared with the total Reads; Reads Map to “-”, the number of Clean Reads that were able to align on the antisense strands of the reference genome data, and the ratio of these reads compared with the total Reads. The number of Mapped Reads in different regions of the reference genome (exon, intron, and intergenic region) was counted, and the distribution of Mapped Reads was drawn. In theory, Reads from mature mRNA should be compared to exon. Reads compared to the introns were the precursor of mRNA. Reads in the intergenic regions were genes not well commented on the genome.*

### Statistics and Annotation of Single Nucleotide Polymorphisms

The SNP site can be divided into transition and transversion according to the different methods of base replacement. Based on the selected reference genome sequence, 3610 SNP sites in the T01 strain, 4057 SNP sites in the T02 strain, and 3797 SNP sites in the T03 strain were demonstrated (Table 3), of which 2439 SNP sites existed in all three strains, 680 SNP sites were unique to the T01 strain, 899 SNP sites were unique to theT02 strain, and 697 SNP sites were unique to the T03 strain (Figure 3B).

**Table 3:** Statistics of the single nucleotide polymorphisms of samples.

BMK-ID	SNP Number	Genic SNP	Intergenic SNP	Transition	Transversion	Heterozygosity
**T01**	3,610	1,795	1,815	41.61%	58.39%	14.96%
**T02**	4,057	1,926	2,131	42.91%	57.09%	15.92%
**T03**	3,797	1,786	2,011	43.35%	56.65%	17.65%

*BMK_ID, the sample number; SNP Number, the number of Single Nucleotide Polymorphisms (SNP): Genic SNP, the number of SNP in coding sequence; Intergenic SNP, the number of SNP intergenic region; Transition, the percentage that the transition SNPS accounted for total SNP sites; Transversion, the percentage that the transversion SNPS accounted for total SNP sites; Heterozygosity, the percentage that the heterozygosity SNPS accounted for the total SNP sites.*

### Discover New Genes

Short peptide chains of less than fifty amino acid residues or containing only an exon were filtered out, and 123 new genes were discovered. Based on the messages from the COG database (Supplementary Figure 1A), twelve new genes were found to participate in carbohydrate transport and metabolism (1), post-translational modification (2), replication (3), energy production and conversion (4), amino acid transport and metabolism (5), translation (6), signal transduction mechanisms (7), general function prediction only(8), and lipid transport and metabolism (9). Seventy-four homologous genes of *B. cinerea*, five homologous genes of *Sclerotinia sclerotiorum*, one homologous gene of *Sclerotinia borealis*, one homologous gene of *Colletotrichum gloeosporioides*, one homologous gene of *Exophiala dermatitidis*, and one homologous gene of *Trichoderma atroviride* were demonstrated in 83 new genes annotated in the Nr database (Supplementary Table 2 and Supplementary Figure 1B).

### Gene Expression and Differential Expression Analyses in Treatments

**FIGURE 4:**
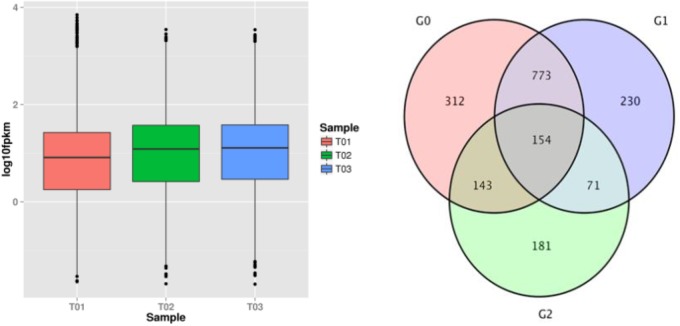
FPKM box plot of the samples and venn of DEGs. The abscissa represents different samples, the ordinate represents the FPKM value of the sample expression. The magenta circle region indicates DEGs that exists in G0(T01_vs_T02); The pale purple circle region indicates DEGs that exists in G1(T01_vs_T03); The pale blue circle region indicates DEGs that exists in G2(T02_vs_T03); The overlapping area indicates DEGs presenced in two or three groups.

The results indicated that 4076 of 10414 genes in the T01 strain, 3962 of 10528 genes in the T02 strain, and 4102 of 10388 genes in the T02 strain were not expressed, while the expression levels of genes in the T02 and T03 strains were higher than those in the T01 strain (Figure 4A), which demonstrated that some genes could be significantly enhanced when the strains were induced by the successive fungicide exposure. Gene expression has temporal and spatial specificity, and the expression levels of gene transcription significantly differing under two different conditions denote DEGs. According to the transcription database, there were 1382 DEGs between the T01 and T02 strains (G0), of which 555 were up-regulated and 827 were down-regulated; there were 1228 DEGs between the T01 and T03 strains (G1), of which 523 were up-regulated and 705 were down-regulated; and there were 549 DEGs between the T02 and T03 strains (G2), of which 294 were up-regulated and 255 were down-regulated. There were 312 unique DEGs in the G0 group of the 927 in the G0 and G1 groups; there were 230 unique DEGs in the G1 group of the 255 in the G1 and G2 groups; and there were 181 unique DEGs in the G2 group of 297 in the G0 and G2 groups. There were 1864 DEGs in total in the G0, G1 and G2 groups, of which 154 were in all three groups (Figure 4B).

### Clustering and Enrichment Analysis of DEGs

**FIGURE 5:**
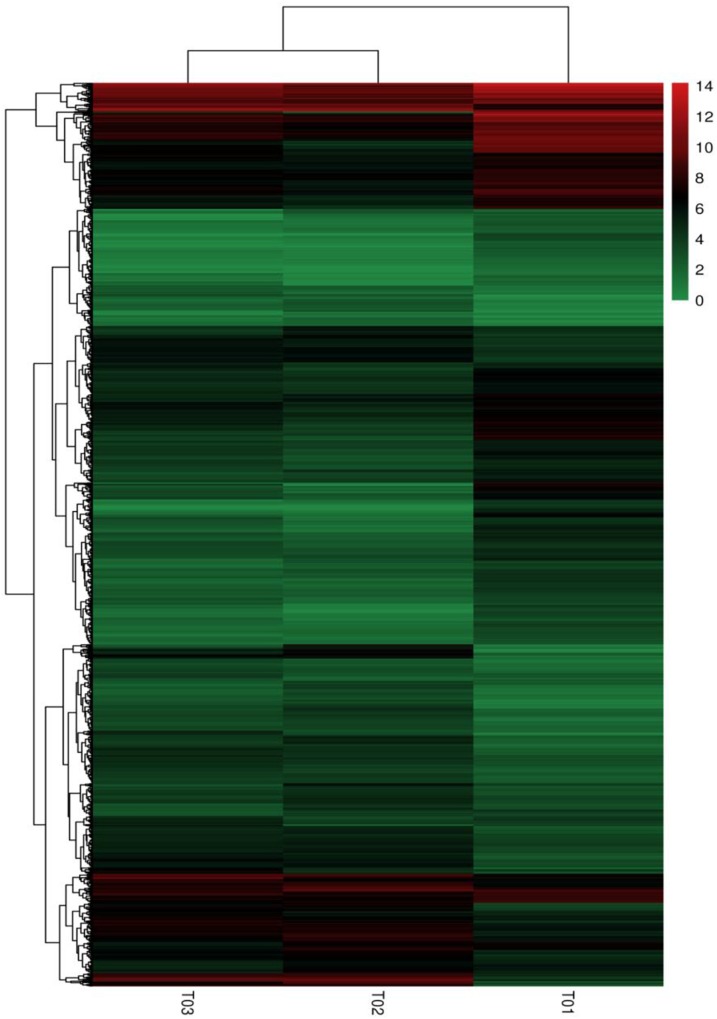
Heat map of the DEGs. The column represents samples, and the row means genes; Color-scaled log 2 (fold change) values for resistant lines, the redder the color is, the higher the gene expression is, and on the contrary, the greener the color is.

**FIGURE 6:**
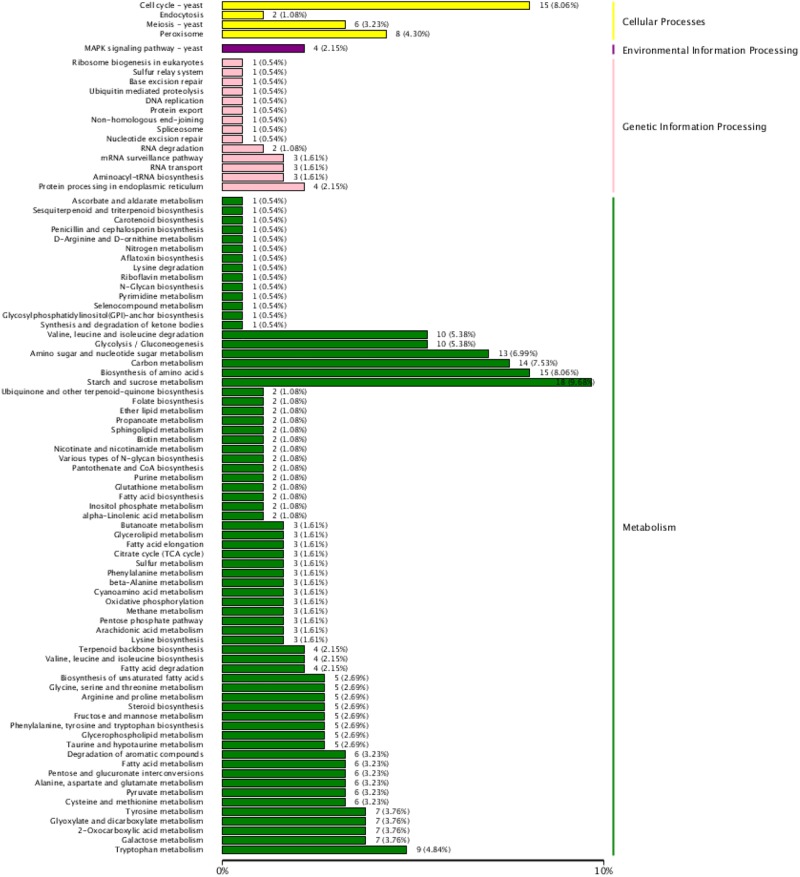
KEEG function classification of the DEGs. The ordinate means KEEG terms, the abscissa means the number of DEGs of each KEEG term. Different color means cellular Processes, environmental Information Processing, genetic Information Processing and metabolism, respectively.

The results shown in an interactive hot diagram indicated that the DEGs in the T02 and T03 strains were less related than those in the T01 and T02 strains or those in the T01 and T03 strains (Figure 5). A total of 196 of 1864 DEGs were annotated in the KEGG database (Figure 6), of which 26 participated in cellular processes; 4 genes participated in MAPK signaling pathway; 23 participated in genetic information processing, 143 participated in metabolism (some genes participated in two or more metabolisms), including 51 participated in amino acid metabolism, 2 in biosynthesis of other secondary metabolites, 59 in carbohydrate metabolism, 10 in energy metabolism, 37 in global and overview maps, 3 in glycan biosynthesis and metabolism, 25 in lipid metabolism, 11 in metabolism of cofactors and vitamins, 6 in metabolism of terpenoids and polyketides, and 3 in nucleotide metabolism.

A total of 1041 of 1864 DEGs were annotated in the COG database (Supplementary Figure 2), including 19 translation, ribosomal structure and biogenesis genes; 26 transcription genes; 31 replication, recombination and repair genes; 3 chromatin structure and dynamics genes; 8 cell cycle control, cell division, chromosome partitioning genes; 14 defense mechanisms genes; 30 signal transduction mechanisms genes; 20 cell wall/membrane/envelope biogenesis genes; 1 cell motility gene; 8 cytoskeleton genes; 2 intracellular trafficking, secretion, and vesicular transport genes; 26 post-translational modification, protein turnover, and chaperones genes; 68 energy production and conversion genes; 132 carbohydrate transport and metabolism genes; 135 amino acid transport and metabolism genes; 6 nucleotide transport and metabolism genes; 26 coenzyme transport and metabolism genes; 64 lipid transport and metabolism genes; 92 inorganic ion transport and metabolism genes; 102 secondary metabolites biosynthesis, transport and catabolism genes; and 211 general function prediction genes only.

### Protein Interaction Network of DEGs

There were 971 and 925 interactions among differentially expressed proteins (DEPs) between the T01 and T02 groups (Table 4) and between the T01 and T03 groups (Supplementary Figure 3), respectively. Mixed-functional oxidase (MFO) genes, including *BC1G_12765*, *BC1G_12768*, *BC1G_16062* and *BC1G-16084*, were obviously upregulated and interacted with DEPs, including *BC1G_06734* and *BC1G_07240* (coenzyme binding and catalytic activity with upregulation) and *BC1G_03483* (coenzyme binding and catalytic activity with down-regulation). There were no significantly upregulated genes in glutathione *S*-transferase and carboxylesterase. *BC1G_04656*, *BC1G_04779*, *BC1G_12366* and *BC1G_13768*, coding transmembrane proteins, and *BC1G_04893*, coding a lysophospholipid enzyme, were obviously upregulated. *BC1G_09151* and *BC1G_14051* [enoyl- (acyl- carrier- protein) reductase (NADH) activity, enoyl-(acyl-carrier-protein) reductase], *BC1G_00543* (monooxygenase activity, FMN binding), and *BC1G_03362* [dihydrolipoyllysine residue (2-methylpropanoyl) transferase activity, cofactor binding] interacted according to the analysis of the protein interaction network.

**Table 4:** Statistics of different interaction of DEPs.

Group	DEPs_tot	DEPs_ act	DEPs_bin	DEPs_cat	DEPs_exp	DEPs_inh	DEPs_ptm	DEPs_rea
T01_vs_T02	971	42	703	83	14	10	31	88
T01_vs_T03	925	61	537	126	18	24	48	111

*Group, DEPs set name; DEPs_tot, the total interaction number of DEPs; DEPs_act, the activation interaction number of DEPs; DEPs_bin, the total binding interaction number of DEPs; DEPs_cat, the total catalysis interaction number of DEPs; DEPs_exp, the expression interaction number of DEPs; DEPs_inh, the inhibition interaction number of DEPs; DEPs_ptm, the ptmod interaction number of DEP; DEPs_rea, the reaction interaction number of DEPs.*

### qRT-PCR of DEGs

According to the transcriptome data, the obvious upregulated expression of four MFO genes, including *BC1G_12765*, *BC1G_12768*, *BC1G_16062*, and *BC1G_16084*; four genes encoding transmembrane proteins, *BC1G_04656*, *BC1G_04779*, *BC1G_12366*, and *BC1G_13768*; four genes encoding Zinc finger proteins acting as regulation factors, including *BC1G_02313 BC1G_04879, BC1G_13764 and BC1G_10483*; and three other important functional genes, including one gene encoding lysophospholipids enzyme *BC1G_04893*, one gene encoding citrate synthase enzyme *BC1G_09151* and one gene encoding gluconolactonase *BC1G_15612*, could be related to the development of resistance of *B. cinerea* against cyprodinil and fenhexamid (Table 5).

**Table 5:** Summary of DEGs between T01_vs_T02 and T01_vs_T03 strains.

Type of gene	Gene name	T01_vs_T02 log2FC	T01_vs_T02 regulated	T01_vs_T03 log2FC	T01_vs_T0 regulated3
**MFO**	*BC1G_12765*	5.032084226	up	5.705700813	up
	*BC1G_12768*	4.589481339	up	4.812436441	up
	*BC1G_16062*	7.332988706	up	6.207540364	up
	*BC1G_16084*	7.346785364	up	5.38584783	up
**Transmembrane protein**	*BC1G_04656*	3.231946068	up	3.377567536	up
	*BC1G_04779*	4.119080357	up	5.474971109	up
	*BC1G_12366*	5.569345676	up	5.05485426	up
	*BC1G_13768*	3.291718093	up	2.738148871	up
**Lysophospholipids enzyme**	*BC1G_04893*	4.311457535	up	6.808033218	up
**Citrate synthase**	*BC1G_09151*	1.557481744	up	1.859216564	up
**Gluconolactonase**	*BC1G_15612*	2.581848717	up	2.322237695	up
**Zinc finger protein**	*BC1G_02313*	2.301844311	up	1.782955337	up
	*BC1G_04879*	3.282655178	up	2.577182495	up
	*BC1G_13764*	1.86468141	up	1.886851742	up
	*BC1G_10483*	2.960218893	up	2.751705456	up

**FIGURE 7:**
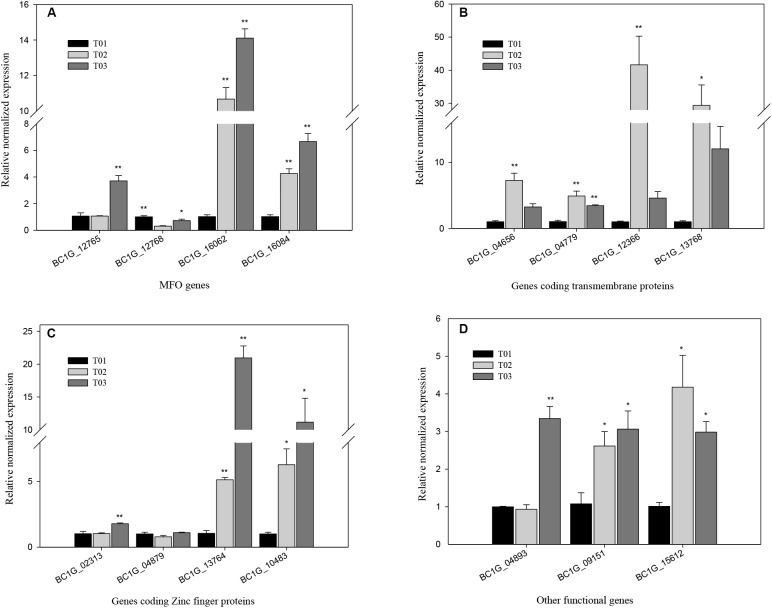
qRT-PCR analysis of significant DEGs in T02 and T03 strains. The asterisk ^∗^ and ^∗∗^ designate statistically significant differences (*P* < 0.05) and extremely significant differences (*P* < 0.01) for each DEG between three strains, respectively. T01, T02, and T03 stand for the BO5.10 strain with no fungicide treatment, F_27_ strains successively screened with EC_50_ concentration of cyprodinil or fenhexamid, respectively. The relative normalized expressions in the DEGs of coding the MFO **(A)**, transmembrane protein **(B)**, Zinc finger proteins **(C)**, and other functional genes **(D)**.

To validate the transcriptome data, 15 significantly up-regulated genes in strains T2 and T3 were analyzed using qRT-PCR (Figure 7). The expression of *BC1G_16062* and *BC1G_16084* in strainsT2 and T3 substantially increased compared with that in the T1 strain (*P* < 0.01), with RNE of 10.7-, 4.3- and 14.1-, 6.7-fold, respectively. However, the expression of *BC1G_12768* was significantly down-regulated in the T2 and T3 strains compared with that in the T1 strain (*P* < 0.05), with RNE of 0.72- and 0.30-fold, respectively (Figure 7A). The RNE of *BC1G_12366* and *BC1G_13768* in the T2 and T3 strains was 41.6-, 29.4- and 4.6-, 12.0-fold (*P* < 0.01), respectively (Figure 7B). The expression of *BC1G_13764* and *BC1G_10483* in the T2 and T3 strains clearly increased the RNE by 5.1-, 6.3- and 21.0-, 11.2-fold, respectively (Figure 7C). The expression of other functional genes, including *BC1G_09151* and *BC1G_15612* in the T2 strain and *BC1G_04893*, *BC1G_09151*, and *BC1G_15612* in the T3 strain, was also enhanced and significantly different from that in the T1 strain (*P* < 0.05) (Figure 7D).

### Prediction Analysis of the Function of Upregulated Genes and Structural Verification

All typical characteristics of the P450 gene, including the sites of heme binding and iron ion binding, monooxygenase activity source, and oxidoreductase activity, were demonstrated in *BC1G_16062, BC1G_1276* and *BC1G_16084*. The MFS_1 domains of *BC1G_13768*, *BC1G_12366*, *BC1G_04779*, and *BC1G_04656* were predicted. Meanwhile the N-terminal ends of the proteins encoded by *BC1G_04779*, *BC1G_12366* and *BC1G_04656* were in the cell, and that of *BC1G_13768* was outside of the cell. Each transmembrane domain (TMD) was linked though the ring protection structure. The reverse repetition structure and typical topological characteristics of the MFS transporter protein were found in each domain. Therefore, *BC1G_12366*, *BC1G_04779* and *BC1G_04656* belonged to the MFS gene (Supplementary Figure 4), which was widely related to multidrug resistance (MDR).

The *cis*-acting elements from 1000 bp upstream to 100 bp downstream of the initiation codons for the significant upregulated genes, including *BC1G_12765*, *BC1G_16062*, *BC1G_16084*, *BC1G_04656*, *BC1G_04779*, *BC1G_12366, BC1G_09151* and *BC1G_04893*, were predicted and found that each had a GC-rich Sp1 element. The Sp1 element was a part of the Zinc-coordinating element and could be regulated by beta-beta-alpha zinc finger proteins. BC1G_02313 (Zn2Cys6), BC1G_04879 (Zn2Cys6), BC1G_10483 (zinc finger, CCHC-type) and BC1G_13764 (Zn2Cys6) were demonstrated to bezinc finger proteins (Supplementary Figure 5). The GAL4 (Zn2Cys6 (or C6 zinc) binuclear cluster of DNA-binding domains was discovered in the *BC1G_02313*, *BC1G_04879* and *BC1G_13764* genes and participated in the regulation of transcription with *cis*-acting elements. However, the ZnF_C2HC domain was found in the *BC1G_10483* gene and participated in the regulation of transcription via *cis*-acting elements. A coiled coil, beta-beta-alpha zinc finger was also anchored on the element. Therefore, we hypothesize that *BC1G_10483* is a very important element in response to pressures from cyprodinil and fenhexamid. Based on the network analysis of protein interaction, there were no DEPs, and the expression of *BC1G_10483* could be regulated by the synergism of multiple transcription factors.

## Discussion

Fungicide resistance has been coming the key problem in crop protection worldwide in the two decades, and has resulted in the greatly decreasing the efficacy, and the increasing concentration to ensure efficacy has produced other problems, such as environment pollution, excessive residue in food, successively strengthening the resistance and increasing the cost of Integrated Pest Management (IPM) strategies (Brent, 2012). Therefore, it is necessary to evaluate the resistance risk in the lab before newer chemicals are wildly put into practice. In this experiment, we continuously screened the B05.10 strain with different concentrations of cyprodinil and fenhexamid and found that the toxicities steadily increased. The cell membrane permeability and glycerinum contents were also significantly increased in those strains whose toxicities have been clearly weakened; in contrast, the oxalic acid (OA) contents markedly decreased. The results were consistent with the early report by Duan et al. (2013). According to the results of the pathway of enrichment analysis, *BC1G_09151*, a speed-limited enzyme of citrate synthase in the tricarboxylic acid (TCA) cycle, was found to be expressed in the T2 and T3 strains and to decrease the oxaloacetic acid content, being is the main precursor in the synthetic process of OA.OA is known to play a very important role in pathogenesis and fungal development (Godoy et al., 1990). Duan et al. (2013) found that when the resistant strain of *S. sclerotiorum* were dealt with fludioxonil, the OA content and virulence of pathogens decreased significantly, then finally resulted in the failure of infection. Meanwhile, *BC1G_15612*, a gluconolactonase gene, was also significantly up-regulated in the glycerol synthetic pathway in the T2 and T3 strains, and its upregulated expression increased the contents of 6-phosphoric acid glucuronic acid and glyceraldehyde 3-phosphate, ultimately leading to the accumulation of glycerinum. In addition, the upregulated expression of *BC1G_13768* promoted the degradation of glycerol phospholipids to glycerophosphate and increased the accumulation of glycerinum in the T2 and T3 strains. Glycerinum is an important factor that can modulate osmotic pressure in cells. In our experiment, the glycerinum contents accumulated in the T2 and T3 strains with the screened pressure of cyprodinil and fenhexamid, whereas the glycerol synthesis rate gradually decreased. This result was consistent with the development of resistance, which was mainly attributed to the enhanced MFO activities to promote the metabolism of fungicides and transmembrane proteins to decreasing fungicide transport into the cell. Some research has demonstrated that fludioxonil interferes with osmotic signal transduction and induces glycerol synthesis in *Neurospora crassa* (Pillonel and Meyer, 1997), *Candida albicans* (Ochiai et al., 2002), and *S. sclerotiorum* (Duan et al., 2013). Our results have suggested that the inhibition of cyprodinil and fenhexamid on B05.10 has induced those strains to produce more glycerinum. At the same time, the changes in the virulence of pathogens in our strains on the host are still worthy of future research.

In recent years, transcriptome analyses has been widely applied in research on fungicidal resistance in some plant pathogens, including *Mycosphaerella graminicola* (Cools et al., 2007), *Fusarium graminearum (*Liu et al., 2010*)*, and *Cucumis sativus* (Wu et al., 2013). Based on our transcriptome data, the expression of genes encoding MFO, transmembrane protein, and zinc finger protein in the T2 and T3 strains was clearly up-regulated through successive treatment with cyprodinil and fenhexamid.

P450 genes are superfamily, which widely distributed in pro- and eukaryotic organisms and were related to the biosynthesis of many biologically important compounds, such as hormones, fatty acids, and sterols (Guengerich, 2004). Becher et al. (2011) reported that the resistance in fungi to azole fungicides mainly relied on the mutation of P450 gene *CYP51* encoding cytochrome P450-dependent sterol demethylase (P450_14αdm_), and resulted in the loss of demethylation activity. In our experiment, RNE of *BC1G_16062* in the T1 strain screened with fenhexamid was found to be significantly promoted. The high homologies of *BC1G_16062* with *CYP37B1* (participating in the metabolism of drugs and steroids in *Caenorhabditis elegans*) (Laing et al., 2012), *BC1G_12765* and *BC1G_16084* with *CYP4* (which played an important role in xenobiotic biotransformation and in modulating the concentrations of eicosanoids during inflammation as well as in the metabolism of clinically significant drugs) (Bylund et al., 2001; Kalsotra and Strobel, 2006) were also demonstrated (Supplementary Figure 6).

The transporter in fungi, removing the intracellular fungicide, is an important resistant mechanism for fungicides and includes two types: one is an ATP binding cassette (ABC), such as CDR genes, and the other is the major facilitator superfamily (MFS), such as the CaMDR genes. ABC transporters are involved in the energy-dependent efflux of sterol demethylation inhibitors (DMIs), which have been described for *Aspergillus nidulans*, *C. albicans*, *M. graminicola*, etc., (Kerr, 2002; Piddockv, 2006). If the charged molecules are unidirectionally pumped as a consequence of the consumption of a primary cellular energy source, electron chemical potential results, which could be used to drive the active transport of additional solutes via secondary carriers (Ren and Paulsen, 2005). *BC1G_13768*, *BC1G_12366*, *BC1G_04779*, and *BC1G_04656* were widely related to multidrug resistance (MDR). The development of MDR based on a single mechanism of resistance, such as the overexpression of genes encoding drug efflux transporters (ABC and MFS), has been observed in isolates of *B. cinerea* resistant to different classes of fungicides (Vermeulen et al., 2001; Moyano et al., 2004; Myresiotis et al., 2007). The expression of the *BcatrD* gene, an ABC transporter gene, is a determinant of the sensitivity of *B. cinerea* to DMI fungicides (Hayashi et al., 2002). The biosynthesis of sterol in *B. cinerea* was stopped by cyprodinil restraining the activity of the 3-keto reductase gene ERG27 in the C-4 methylation process (Albertini and Leroux, 2004). Fenhexamid inhibits the activity of cystathionine β-lyase and hinders the biosynthesis of methionine (Leroux et al., 2002). According to the data analysis, the SNP sites encoding the genes of the 3-keto reductase gene ERG27 and cystathionine β-lyase were not found in the T02 and T03 strains. Therefore, we hypothesized that the B05.10 strain screened with cyprodinil and fenhexamid contained base mutations at target points.

Sp1 is a stress response element of −50 bp in cells and plays a key role in adapting to adverse ecological conditions, including the selective pressure of fungicides (Chang et al., 2017). SP1 can modulate the expression of surviving cells through the MAPK signaling pathway to regulate the drug resistance of leukemia stem cells (Zhang et al., 2015). It is a transcription activating factor of zinc finger-rich glutamine that regulates the expression of target genes involved in transcription regulatory factors, contains the structure of the zinc finger in the carboxyl end and especially combines the GC box in DNA promoter (Pearson et al., 2008).The overexpression of EGR1 (zif268, zinc fingers) was able to activate a construct containing tandem of the MDR1 promoter at SP1 sites and increased its expression in human acute and chronic leukemias (McCoy et al., 1995). The active zone of the zinc finger exists in the middle, participates in regulating the expression in the target genes and combines the other transcription factors. The combination ability between zinc finger and DNA, the activity of transcription and modulation domain and the content of zinc finger in cells have played a key role in the response to external stress factors. The zinc finger protein of MSN2 or MSN4 acts as a transcriptional activator in *Saccharomyces cerevisiae* to activate transcription via response to stress (Fabrizio et al., 2004), and the zinc finger protein of seb1 also responds to high osmotic stress in *Trichoderma atroviride* (Peterbauer et al., 2002).

## Conclusion

The toxicities, conductivities and glycerinum contents of the *B. cinerea* BO5.10 strain with the successive pressure of cyprodinil and fenhexamid increased. Some key genes encoding MFO, transmembrane proteins, zinc finger proteins, citrate synthase enzyme, gluconolactonase, and lysophospholipid enzymes have been significantly upregulated, and their functional prediction analysis indicated that *BC1G_10483* was a ZnF_C2HC transcriptional regulator by means of interaction with the Sp1 element of these genes in response to pressures from cyprodinil and fenhexamid. Our conclusions were necessarily tenuous and further studies were required since the information about the resistant level of *B. cinerea* on traditional chemicals, and whether cross-resistance between those chemicals and the newer tested fungicides or not were not clearly. However, all results could contribute to a better understanding of the resistance mechanism of *B. cinerea* on cyprodinil and fenhexamid and provided a foundation for designing the IPM strategy for the effectively controlling gray mold in the field.

## Author Contributions

CG conceived and designed the experiments. CG, YZ, LS, and XW performed the experiments. XW and CG wrote and revised the paper. All authors approved the final version of the manuscript.

## Conflict of Interest Statement

The authors declare that the research was conducted in the absence of any commercial or financial relationships that could be construed as a potential conflict of interest.

## Footnotes

1 https://www.ncbi.nlm.nih.gov/bioproject/15632

2 https://myhits.isb-sib.ch/cgi-bin/motif_scan

3 http://prodom.prabi.fr/prodom/current/html/home.php

4 http://www.enzim.hu/hmmtop

5 http://gene-regulation.com/pub/programs/alibaba2/index.html

6 http://jaspardev.genereg.net/

7 http://ftfd.snu.ac.kr/blast.php

8 http://smart.embl.de/

9 https://pfam.xfam.org/search
