# Supplementary material for: Transcriptome and Resistance-Related Genes Analysis of Botrytis cinerea B05.10 Strain to Different Selective Pressures of Cyprodinil and Fenhexamid
Source: Front Microbiol. 2018 Oct 30;9:2591. doi: 10.3389/fmicb.2018.02591 (PMC6218599; doi:10.3389/fmicb.2018.02591)
Supplement: Supplementary file 1 [file Data_Sheet_1.docx]

**(A)
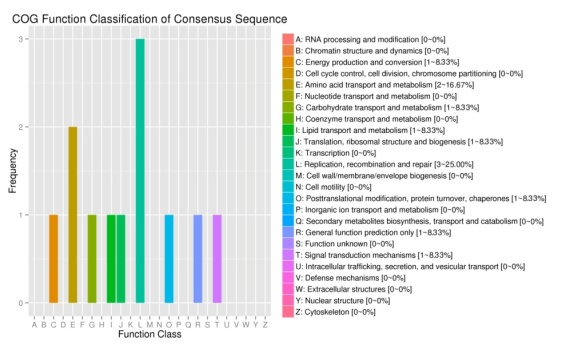
**

**(B)
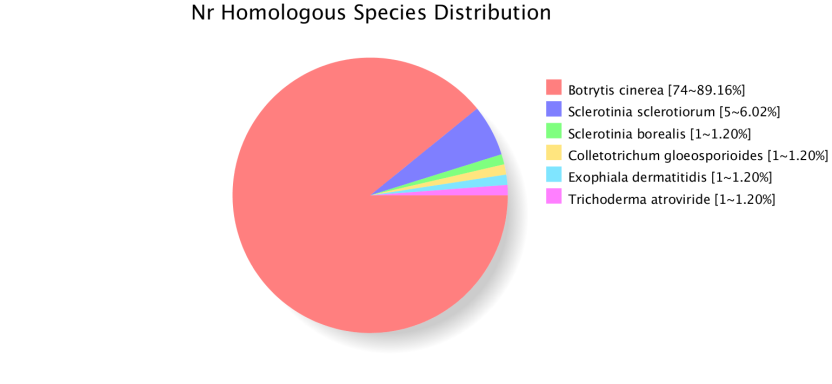
**

**FIGURE 1 COG function classification of consensus sequence and Nr homologous species distribution**. The abscissa means COG terms, ordinate means the frequency of consensus sequences of each COG term in all the DEGs. Different color means different COG term, respectively. The consensus sequences are compared to different species, and the region size is determined by the percentage of sequences mapped to the corresponding species in all consensus sequences.

**
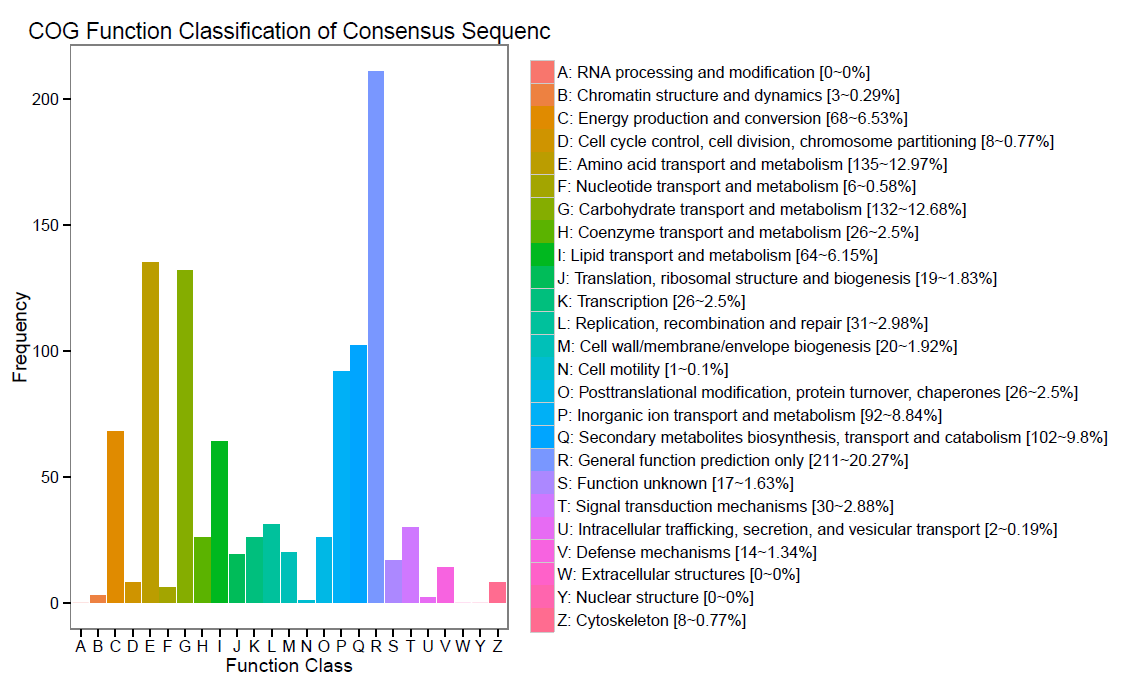
**

**FIGURE 2 COG function classification of the DEGs.** The abscissa means COG terms, ordinate means the frequency of DEGs of each COG term in all the DEGs. Different color means different COG term, respectively.

**
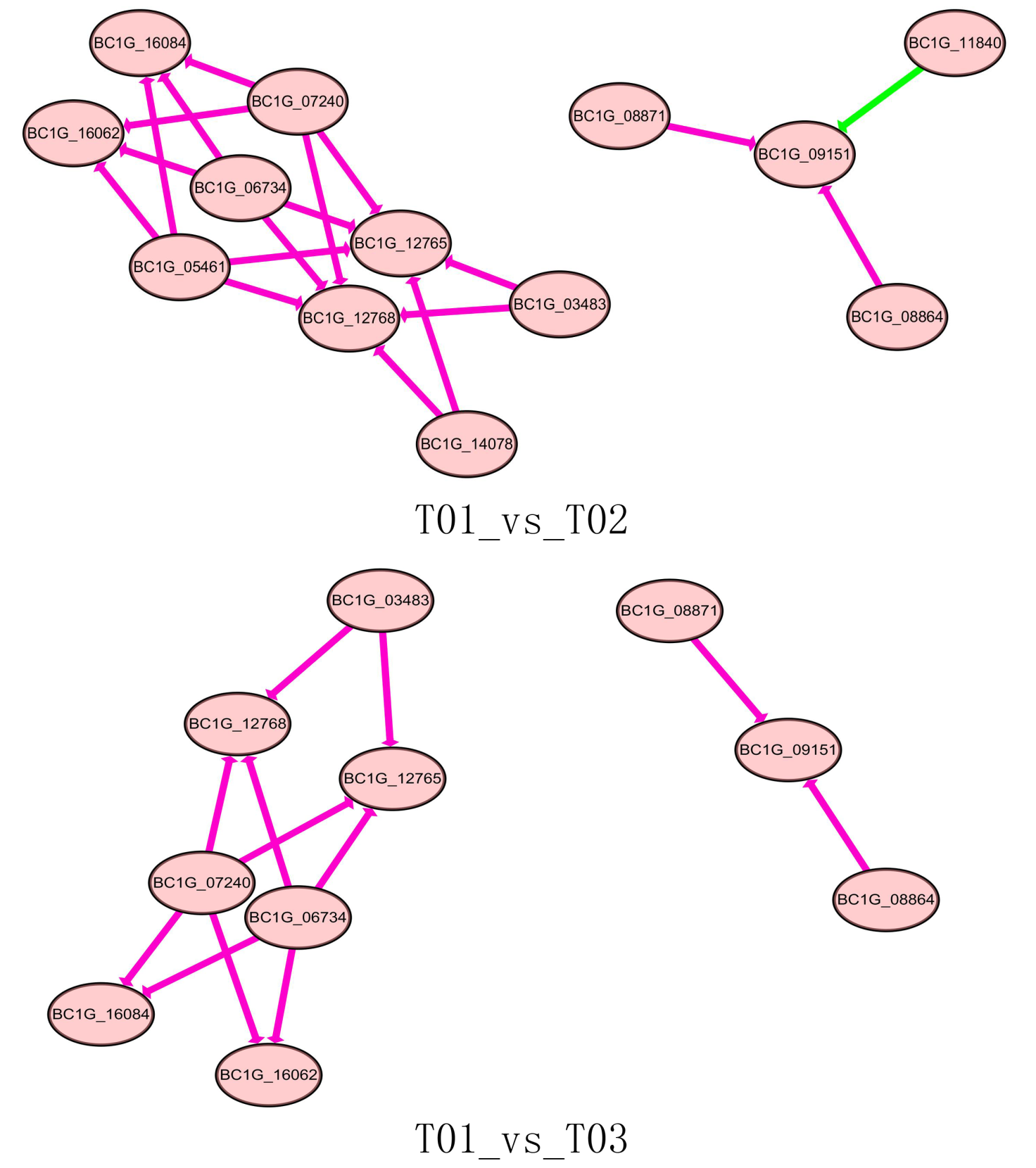
**

**FIGURE 3 Network diagram of protein interaction of the DEGs betweenT01 and T02 groups, T01 and T03 groups.** The direction of arrow indicated source proteins to target proteins;.The Rose red line, the mode between proteins was activation; And the blue solid line, the mode between proteins was reaction.

**
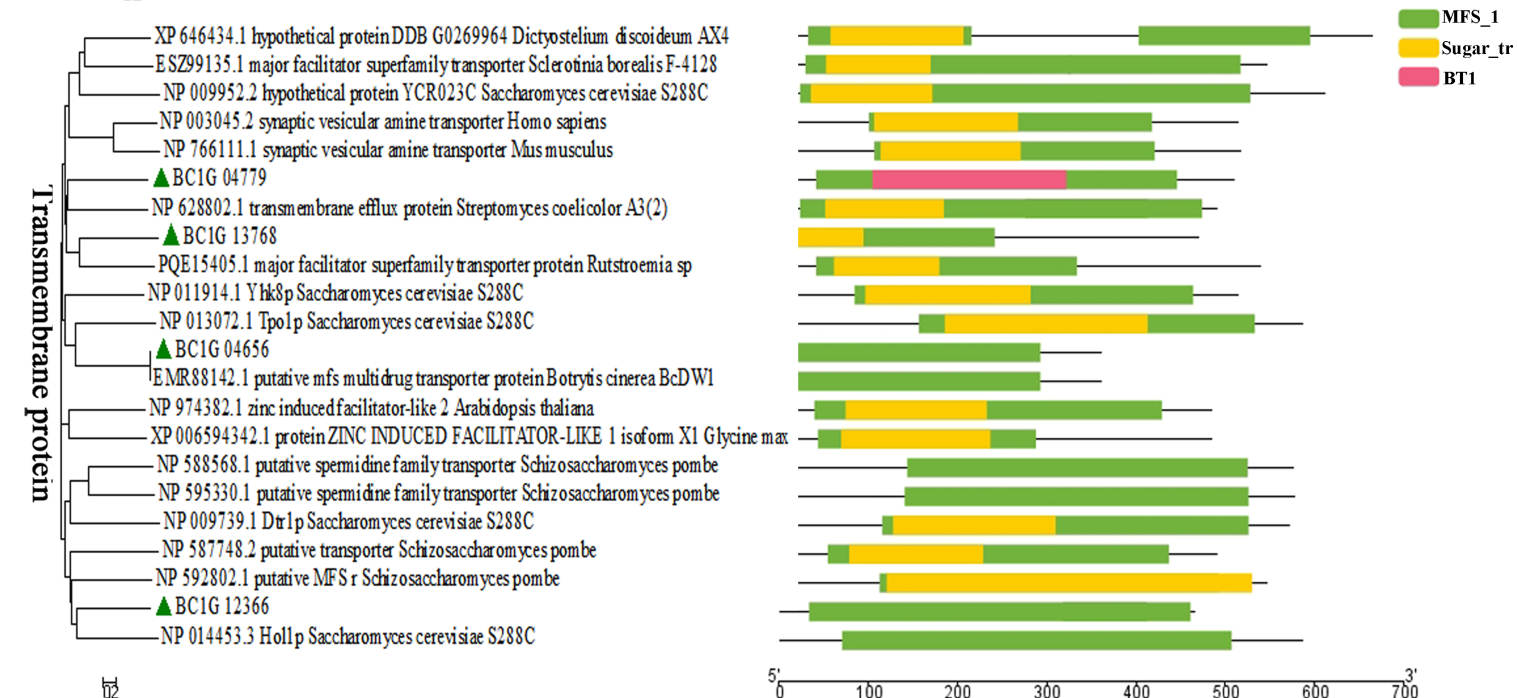
**

**FIGURE 4 Phylogenetic tree and structural domain of transmembrane protein of *Botrytis cinerea* and other species by the Neighbor-joining method and the PFAM SEARCH**. **MFS-1**, as well as **Sugar_tr** and **BT1** are members of the major facilitator superfamily, which is one of the two largest families of membrane transporters found on Earth; There was high homologies between BC1G*_*04656 and BcDW1, which might participate in the transport of multidrug in *Botrytis cinerea*


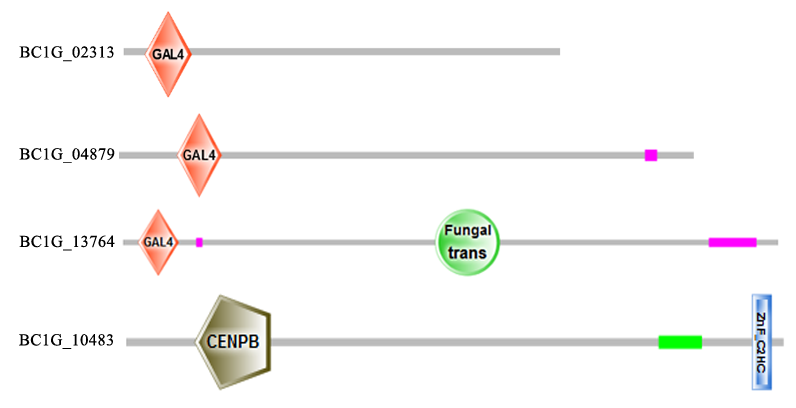


**FIGURE 5 Structural domain of Zinc finger proteins**. GAL4, GAL4-like Zn(II)2Cys6 (or C6 zinc) binuclear cluster DNA-binding domain; the purple box: a region of low compositional complexity, as detected by the SEG program; Fungal specific transcription factor domain: included transcriptional activator xlnR, yeast regulatory protein GAL4, and other transcription proteins regulating a variety of cellular and metabolic processes; **CENPB:** The CENPB-type HTH domain is a DNA-binding, helix-turn-helix (HTH) domain of about 70-75 amino acids, present in eukaryotic centromere proteins and transposases; the *green box:* a coiled coli region, as detected by the COILS program; **ZnF_C2HC** domain: This entry represents the CysCysHisCys (CCHC) type zinc finger domains, and have the sequence:C-X2-C-X4-H-X4-C.


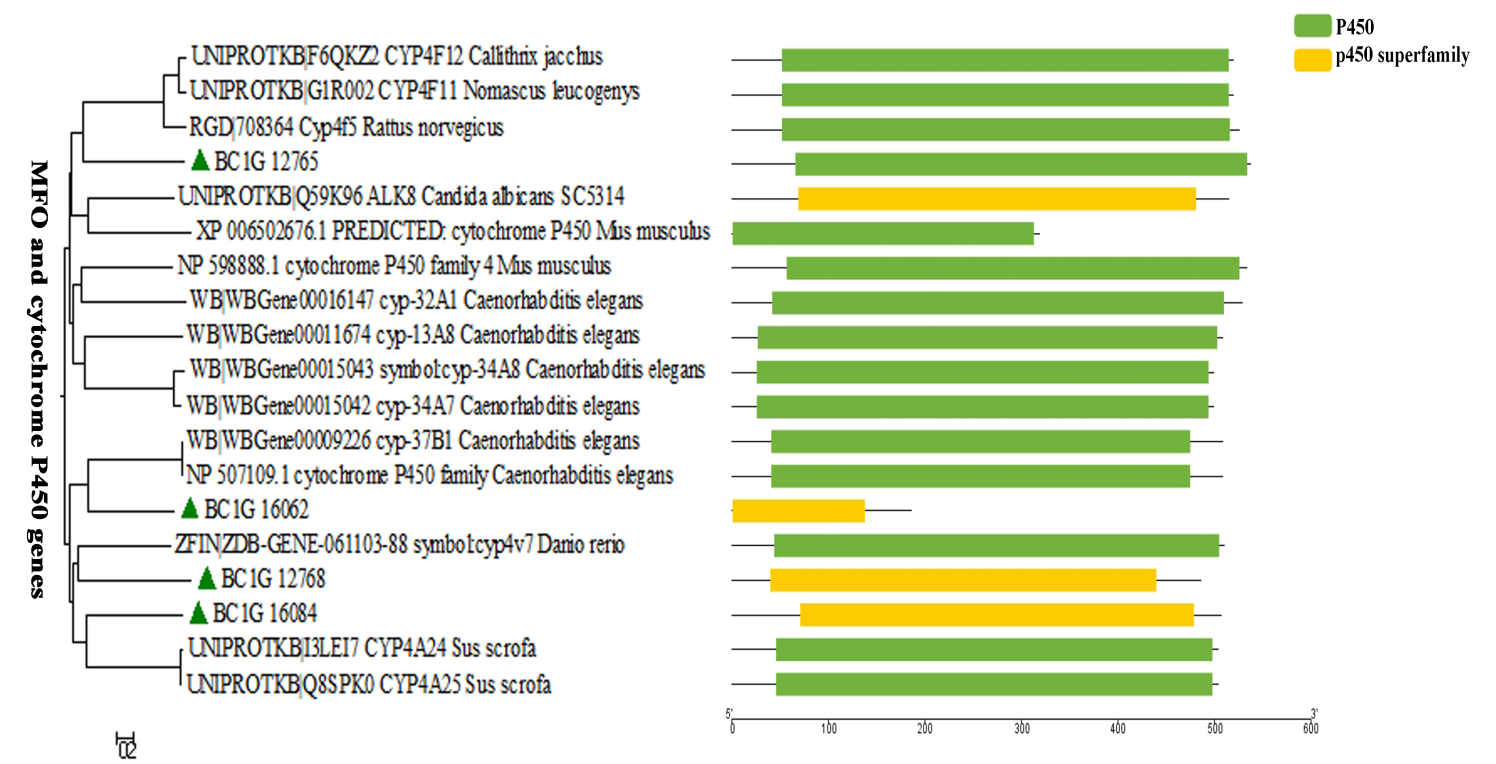


**FIGURE 6 Phylogenetic tree and structural domain of MFO genes of *Botrytis cinerea* and cytochrome P450 genes of other species by the Neighbor-joining method and the PFAM SEARCH**. **P450** and **p450 superfamily** are haem-thiolate proteins involved in the oxidative degradation of various compounds, such as environmental toxins and mutagens.
