# Supplementary material for: Transcriptome and Resistance-Related Genes Analysis of Botrytis cinerea B05.10 Strain to Different Selective Pressures of Cyprodinil and Fenhexamid
Source: Front Microbiol. 2018 Oct 30;9:2591. doi: 10.3389/fmicb.2018.02591 (PMC6218599; doi:10.3389/fmicb.2018.02591)
Supplement: Supplementary file 2 [file Data_Sheet_2.docx]

**TABLE 1 Primer of significant up-expression for qRT-PCR**

| Gene | Prime | Squence（5’-3’） | Length |
| --- | --- | --- | --- |
| *β_tubulin* | *Tubulin* -F | GAGCTGTTTTCCCTTCCATTGTC | 146 |
|  | *Tubulin* -R | GACGAC ACCGTGCTCGATTGG |  |
| *BC1G_12765* | *BC1G_12765*-F | ATGCAATCAAGCAGGTATGATAGA | 133 |
|  | *BC1G_12765*-R | CCTGACCATCCACAACTTCG |  |
| *BC1G_12768* | *BC1G_12768*-F | TGCTCAAGGTATGTTACTTGGACT | 90 |
|  | *BC1G_12768*-R | CACGCATCGCCTGTGTTTTT |  |
| *BC1G_16062* | *BC1G_16062*-F | CGGCCTCAAGTAAGATTCCCT | 111 |
|  | *BC1G_16062*-R | GGTAGGTCTCCATCCAAGCG |  |
| *BC1G_16084* | *BC1G_16084*-F | AGTTCACATTTCCACAATCAAAACG | 82 |
|  | *BC1G_16084*-R | AGCTCATCAGGGGCTATTCG |  |
| *BC1G_04656* | *BC1G_04656*-F | CGGAGTTTCAGTGTTCCTGC | 81 |
|  | *BC1G_04656*-R | CATCTCCGAAACTCGCCCTA |  |
| *BC1G_04779* | *BC1G_04779*-F | TCGCCATCCCCAAGTAATCG | 89 |
|  | *BC1G_04779*-R | AGGATCGTGGTCGCCATTTT |  |
| *BC1G_12366* | *BC1G_12366*-F | TACTTACGGGCAGCAACCAT | 95 |
|  | *BC1G_13768*-R | ACTGACTGATTGCGTGCTTC |  |
| *BC1G_13768* | *BC1G_13768*-F | TGAATAGCAGACCAGCACACA | 82 |
|  | *BC1G_13768*-R | CCAAACATTTTCTCGCCGGA |  |
| *BC1G_04893* | *BC1G_04893*-F | ATAACTGAGAGGTGTTTCTCGCA | 84 |
|  | *BC1G_04893*-R | TACGCGAATTTTTGGGTCGTC |  |
| *BC1G_09151* | *BC1G_09151*-F | GAGTGCTGAAACAGCCATCG | 109 |
|  | *BC1G_09151*-R | GAGTGCTGAAACAGCCATCG |  |
| *BC1G_15612* | *BC1G_15612*-F | GGCAAGGAATCCGTCCTCAA | 153 |
|  | *BC1G_15612*-R | TTCCGTCGGTAATGTAGGCG |  |
| *BC1G_02313* | *BC1G_02313-*R | TATCACTTATCCCGTGCCGC | 180 |
|  | *BC1G_02313-*F | GAGATCGGTATCCGCAGGTG |  |
| *BC1G_04879* | *BC1G_04879-*R | CCTTAACCCACATCGCTGGT | 170 |
|  | *BC1G_04879-*F | GCGTGTAGTATTTGCCGCAG |  |
| *BC1G_13764* | *BC1G_13764-*R | TCAGCGATACCCGCATTACC | 194 |
|  | *BC1G_13764-*F | AGATTTGTGGGCGAGTCGTT |  |
| *BC1G_10483* | *BC1G_10483-*R | GCCCAATCCGCAGGAAAATC | 151 |
|  | *BC1G_10483-*F | GCAAGGCAATACAACCAGGC |  |

**TABLE 2 Statistics of the functional annotation of new genes in different databases**

| **#Anno_Database** | **Annotated_Number** | **300<=length<1000** | **length>=1000** |
| --- | --- | --- | --- |
| COG_Annotation | 12 | 8 | 4 |
| GO_Annotation | 31 | 17 | 14 |
| KEGG_Annotation | 15 | 11 | 4 |
| Swissprot_Annotation | 20 | 10 | 10 |
| nr_Annotation | 83 | 35 | 48 |
| All_Annotated | 83 | 35 | 48 |
